# Impact of SARS-CoV-2 vaccination on FcγRIIIA/CD16 dynamics in Natural Killer cells: relevance for antibody-dependent functions

**DOI:** 10.3389/fimmu.2023.1285203

**Published:** 2023-11-16

**Authors:** Cristina Capuano, Davide De Federicis, Daniel Ciuti, Ombretta Turriziani, Antonio Angeloni, Emanuela Anastasi, Giuseppe Giannini, Francesca Belardinilli, Rosa Molfetta, Domenico Alvaro, Gabriella Palmieri, Ricciarda Galandrini

**Affiliations:** ^1^ Departmental Faculty of Medicine and Surgery, UniCamillus-Saint Camillus International University of Health and Medical Sciences, Rome, Italy; ^2^ Department of Experimental Medicine, Sapienza University of Rome, Rome, Italy; ^3^ Department of Molecular Medicine, Sapienza University of Rome, Rome, Italy; ^4^ Department of Translational and Precision Medicine, Sapienza University of Rome, Rome, Italy

**Keywords:** SARS-CoV-2 vaccine, NK cells, CD16, ADCC, IFNγ, memory NK cells, CD16 polymorphism

## Abstract

**Introduction:**

Natural Killer (NK) cells contribute to the protective effects of vaccine-induced antibodies thanks to the low affinity receptor for IgG, FcγRIIIA/CD16, whose aggregation leads to the killing of infected cells and IFNγ release, through which they potentiate adaptive immune responses.

**Methods:**

Forty-seven healthy young individuals undergoing either homologous (ChAdOx1-S/ChAdOx1-S) or heterologous (ChAdOx1-S/BNT162B2) SARS-CoV-2 vaccination settings were recruited. Peripheral blood samples were collected immediately prior to vaccination and 8 weeks after the booster dose. The phenotypic and functional profile of NK cells was evaluated by flow cytometry at both time points. Serum samples were tested to evaluate circulating anti-Spike IgG levels and cytomegalovirus serostatus. CD16 F158V polymorphism was assessed by sequencing analysis.

**Results:**

The downregulation of CD16 and the selective impairment of antibody-dependent cytotoxicity and IFNγ production in CD56^dim^ NK population, persisting 8 weeks after boosting, were observed in heterologous, but not in homologous SARS-CoV-2 vaccination scheme. While the magnitude of CD16-dependent functions of the global CD56^dim^ pool correlated with receptor levels before and after vaccination, the responsivity of NKG2C^+^ subset, that displays amplified size and functionality in HCMV^+^ individuals, resulted intrinsically insensitive to CD16 levels. Individual CD16 responsiveness was also affected by CD16F158V polymorphism; F/F low affinity individuals, characterized by reduced CD16 levels and functions independently of vaccination, did not show post-vaccinal functional impairment with respect to intermediate and high affinity ones, despite a comparable CD16 downregulation. Further, CD16 high affinity ligation conditions by means of afucosylated mAb overcame vaccine-induced and genotype-dependent functional defects. Finally, the preservation of CD16 expression directly correlated with anti-Spike IgG titer, hinting that the individual magnitude of receptor-dependent functions may contribute to the amplification of the vaccinal response.

**Conclusion:**

This study demonstrates a durable downmodulation of CD16 levels and Ab-dependent NK functions after SARS-CoV-2 heterologous vaccination, and highlights the impact of genetic and environmental host-related factors in modulating NK cell susceptibility to post-vaccinal Fc-dependent functional impairment.

## 1 Introduction

Antibodies (Abs) are a critical component of the protective immune response elicited by vaccination. Upon SARS-CoV-2 vaccination, the titer of neutralizing receptor binding domain (RBD)-specific Abs correlates with vaccine efficacy (1, 2). However, accumulating evidence point to a key role for Fc-mediated effector functions in sustaining protection against severe disease in different contexts, such as antigenically shifted viral variants, immunocompromised individuals, or time-dependent waning of neutralizing Abs (3–9). Indeed, interaction of the conserved Fc region of IgG with FcγRs or complement can promote antibody-dependent Natural Killer (NK) cell activation (ADCC), antibody-dependent cellular phagocytosis by professional phagocytes and complement-dependent cytotoxicity (3–9).

NK cells constitute a heterogeneous lymphocyte component of innate immunity, playing an important role in the control of infections and cancers (10, 11). In humans, NK cells mediate Fc-dependent Ab functions thanks to the expression of FcγRIIIA/CD16, a type I, low affinity, activating receptor that recognizes IgG-opsonized cells or multimeric IgG-containing immune complexes; it associates with homo- or heterodimers of FcεRIγ and CD3ζ adaptors which connect the receptor to intracellular signaling pathways (12, 13).

CD16 represents the prototype of NK activating receptors; it is expressed on the vast majority of mature CD56^dim^ NK cells and its engagement unleashes the full spectrum of NK cell functions against intracellular pathogens, by promoting the killing of infected cells, as well as the production of pro-inflammatory cytokines and chemokines, including IFNγ, TNFα, IL-6, GM-CSF and CCL5, through which they interface with and potentiate adaptive immune responses (14, 15). IFNγ stands as a well-recognized key immunoregulatory factor in the shaping of adaptive immune responses, by modulating dendritic cell (DC) maturation and effector T cell development (14–17); additionally, IFNγ represents the major factor that promotes immunoglobulin class switch toward IgG isotype (18–20). Hence, NK cells can play a role in the amplification of anti-pathogen immune response; on the other hand, the interaction of IgG with CD16 may be critical in dictating the efficacy of vaccination-induced Abs and of mAb-based regimens in infectious diseases.

Differently from all the other innate immune cells, NK cells do not co-express the inhibitory FcγRIIb isoform, and are thus extremely sensitive to Ab-dependent activation; the amplitude of FcR-dependent functions is regulated by genetic and environmental factors affecting the avidity of Fc-CD16 interaction, as well as receptor signaling capability.

IgG glycosylation, for instance, is a post-translational modification with profound implications on Fc effector functions. Recently, the correlation between afucosylated anti-Spike IgG, that possess a greatly increased affinity for CD16, and Covid-19 severity has been evidenced, corroborating the idea that NK cells may participate to pro-inflammatory pathogenic signature (21). Additionally, heightened fucosylation and sialylation of vaccine-induced Abs may contribute to the quality of FcR-mediated protection, by impacting the efficiency of Fc effector functions (5, 22).

A single nucleotide polymorphism of the *FCGRIIIA* gene impacts CD16 affinity and expression levels by determining the presence of valine or phenylalanine at position 158. FcγRIIIA-158V high-affinity variant may associate with improved biological responses to mAb-based therapies and with Ab-mediated inflammatory signature (23, 24). Conversely, this same polymorphism correlated with HIV disease progression (25) and lack of protection in HIV VAX004 vaccine trial (26). Moreover, FcγRIIIA-158V-dependent enhanced ADCC is a hallmark of severe Covid-19 (27).

In the context of the heterogeneity of the human NK cell pool, a particular subset of mature CD56^dim^ cells, characterized by expression of CD94/NKG2C activating receptor specific for HLA-E, is worth noting. NKG2C^+^ NK cells expanding in HCMV seropositive individuals, named memory or adaptive, are highly specialized towards Ab-dependent responses (28–30). Memory NK cells are marked by the absence of FcεRIγ adaptor protein (28–32), which is mechanistically linked to CD16 enhanced responsiveness, possibly due to its exclusive association to CD3ζ homodimer (33); indeed, memory NK cells are endowed with a greatly enhanced ability to produce IFNγ in a CD16-dependent manner (31, 32, 34), also thanks to the hypo-methylated state of *IFNG* regulatory region (35), thus providing a prompt and powerful response against antibody-opsonised virus-infected cells. Notably, in the context of SARS-CoV-2 and Influenza virus vaccination, the frequency of NKG2C^+^ NK cells at baseline, or their expansion, have been correlated with increased vaccinal response (36, 37).

CD16 effector functions are also regulated by receptor expression levels. Indeed, cytokine-induced activation, or CD16 ligation by opsonizing IgG, induce metalloproteinase-mediated receptor shedding (38, 39); further, lysosomal targeting of CD16-associated signaling elements follows its chronic engagement and internalization *in vitro* (40, 41). The impact of diminished expression of surface CD16 on the avidity of Fc-CD16 interaction, together with the initiation of negative intracellular regulatory circuits upon sustained receptor ligation, lead to NK hyporesponsiveness to further stimulation (42).

Notably, previous evidence highlighted a persistent downregulation of CD16 expression levels upon Influenza virus vaccination, arguing that vaccine-elicited antigen-antibody complexes may provide chronic CD16 engagement *in vivo* (43). It is worth noting that SARS-CoV-2 Spike protein was reported to persist in secondary lymphoid organs and/or systemically following administration of the mRNA vaccine formulation (44, 45), suggesting that NK cells are chronically exposed to immune complexes.

Several evidence revealed that NK cell compartment is quantitatively and qualitatively perturbed upon vaccination (46). A rapid drop of CD16^+^ NK cells and the augmentation of IFNγ-producing NK cells were reported in SARS-CoV-2 vaccinees, and, together with a higher frequency of NKG2C^+^ NK cells at baseline, these events correlated with vaccine outcome, in terms of antibody response durability and breadth against emerging virus variants (36, 47, 48).

In such context, a deep analysis of CD16 dynamics and its functional correlates is lacking. Here we report an *ex vivo* and *in vitro* characterization of the selective impact of heterologous (adenoviral prime followed by mRNA boost platforms) SARS-CoV-2 vaccination setting on CD16 expression and functional outcome.

We also address the possible impact of CD16 allelic variants and of HCMV serostatus on post-vaccination Fc-dependent responses.

## 2 Materials and methods

### 2.1 Cohort and study design

Forty-seven healthy young individuals (age range 22-35y) were recruited in the study and received a first dose of ChAdOx1-S (Vaxzevria, Astra Zeneca) adenovirus-based vaccine. After 10 weeks, 10 individuals received the booster dose in homologous setting and 37 received BNT162b2 mRNA-based vaccine (Comirnaty, Pfizer/BioNTech) in heterologous setting. Serological, demographic and genetic features of vaccinees are reported in **Supplementary Table 1**.

All procedures involving human participants were in accordance with the ethical standards of the institutional research committee and with the Declaration of Helsinki and its later amendments or comparable ethical standards.

This study was approved by the Ethics Committee of Sapienza University of Rome (EC identifier 6020 - Prot. 0486/2021). All vaccinees gave written informed consent to the study.

### 2.2 Peripheral blood mononuclear cell isolation

Heparinized venous blood samples were collected before vaccination (T0) and 8 weeks after boost (T1). Blood was diluted 1:1 in Phosphate Buffered Saline (PBS) (Euroclone, Italy) and mononuclear cells were isolated by Ficoll-Hypaque (Lymphoprep, Euroclone) density gradient centrifugation PBMCs were resuspended in a solution of 90% Fetal Calf Serum (FCS) (HyClone; Euroclone) and 10% dimethyl sulfoxide (Merck, Germany), and cryopreserved at -80°C until use. T0 and T1 paired samples were thawed and cells were let to recover over-night at 37°C in 24-well plates at 2x10^6^ cells/ml in 10% FCS, 1% L-glutamine (Euroclone)- and 1% penicillin/streptomycin (Euroclone)-containing RPMI 1640 medium, before proceeding to phenotypic and functional analysis in parallel. Cell viability, evaluated by trypan blue exclusion, was comparable in paired T0 and T1 samples.

### 2.3 Cell lines

The following human cell lines were used as targets: CD20^+^ lymphoblastoid Raji, provided by Dr. F.D. Batista (Ragon Institute of MGH, MIT and Harvard, Cambridge, MA, USA) and K562 erythroleukemia were kept in culture for less than two consecutive months in 10% FCS- and 1% L-glutamine-containing RPMI 1640 medium, and routinely tested for mycoplasma contamination by EZ-PCR Mycoplasma test kit (Biological Industries, Israel, 20-700-20). Cells were authenticated (last testing July 2022) by morphology, growth, immunophenotypic characteristics and biological behavior.

### 2.4 Cell stimulation and functional assays: degranulation and IFNγ production

To determine CD16-mediated CD107a (LAMP-1) surface mobilization and/or intracellular IFNγ production, PBMCs (8x10^5^ cells/sample) were allowed to interact with CD20^+^ Raji target (effector: target ratio, 2:1) opsonized or not with the minimum saturating dose (1 μg/1x10^6^ cells) of the chimeric IgG1k type I anti-CD20 rituximab or, where indicated, with 0.1 μg/1x10^6^ cells of humanized IgG1k type II anti-CD20 afucosylated obinutuzumab (kindly provided by Dr. Christian Klein, Roche Innovation Center Zurich, Schlieren, Switzerland). Samples were centrifuged for 1 minute at 600 rpm to allow conjugate formation, and incubated for 6 hours at 37°C in the presence of 50 μM Monensin (Golgi-stop Merck, M5273). After the first hour, 10 μg/ml of Brefeldin A (Merck, B7651) was added to each sample. At the end of stimulation, cells were diluted in 2% FCS- and 2 mM EthyleneDiamineTetraAcetic acid disodium salt (EDTA)-containing PBS (PBS-EDTA) to promote conjugate disruption. In a group of individuals, selected on the basis of higher cell recovery, IFNγ production and degranulation in ADCC and natural stimulation settings were assessed in parallel. To assess natural effector functions, PBMCs (8x10^5^ cells/sample) were stimulated with K562 sensitive target (effector: target ratio, 6:1). To this end, PE-conjugated anti-CD107a mAb (BD Biosciences, USA clone: H4A3, 555801) was added at the beginning of the test.

### 2.5 Immunostaining and FACS analysis

Surface staining of PBMC samples was performed with saturating concentrations of the following fluorochrome-conjugated mAbs: anti-CD3 PerCP-Vio700 (Miltenyi Biotec, clone: BW264/56, 130-113-132), anti-CD56 APC-Vio770 (Miltenyi Biotec, clone: REA 196, 130-114-548), anti-CD16 PE-Vio770 (Miltenyi Biotec, clone: REA 423, 130-113-394), anti-NKG2C-PE (Miltenyi Biotec, clone: REA 205, 130-119-776), for 30 minutes at 4°C, washed with PBS-EDTA (used for all washing steps) and fixed with 2% paraformaldehyde (Merck) for 16 minutes at room temperature.

For intracellular IFNγ detection, samples were permeabilized by incubating with PBS-EDTA supplemented with 0.05% Triton-X 100 (Biorad) for 22 minutes at room temperature, centrifuged at 1600 rpm at 4°C for 5 minutes, incubated with APC-conjugated anti-IFNγ (BD Biosciences, clone: B27, 554702) mAb for 30 minutes, washed and subjected to cytofluorimetric analysis. Where required, twin samples were stained with isotype control mAb, to set the threshold for antigen positivity. All samples were acquired with a FACSCanto II (BD Biosciences), and data were analysed with FlowJo 10.8.2 (BD Biosciences) software. NK cell subsets were identified by antibody combinations within the lymphocyte region, determined by FSC and SSC physical parameters.

### 2.6 Analysis of CD16 polymorphisms

Genomic DNA was extracted from PBMCs using the QIAamp DNA Blood mini Kit (Qiagen, Germany) according to manufacturer’s instructions, and the region which contains the polymorphic sequence was amplified by PCR, using the following primers: forward 5’-CCCTTCACAAAGCTCTGCACT-3’; reverse 5-ATTCTGGAGGCTGGTGCTACA-3’; sequencing: 5-CCCCAAAAGAATGGACTGAA-3’. Sequencing reaction was performed using the BigDye Terminator v3.1 Cycle Sequencing Kit and a SeqStudio™ Genetic Analyzer System (Thermo Fisher Scientific, USA). Sequences were finally analysed with the Sequencing Software Analysis 7 (Thermo Fisher Scientific) (41, 49).

### 2.7 Serological analysis

HCMV serostatus was assessed by quantification of plasma anti-HCMV IgG levels, using CMV IgG Immulite 2000 System (Siemens Healthineers, Italy). HCMV seropositivity corresponded to >1 arbitrary units (au), according to manufacturer’s instructions, and as previously reported (34).

The level of circulating anti-Spike IgG Abs was assessed in serum samples using the LIAISON^®^ SARS-CoV-2 Trimeric S IgG assay (DiaSorin SpA, Italy), according to manufacturer’s instructions. Antibody titers were expressed in Binding Antibody Units/ml (BAU/ml), with a quantification range of 4.81 to 2080 BAU/ml and 33.8 BAU/ml cut-off value.

The detection of circulating anti-Nucleoprotein (N) IgM, IgG, and IgA Abs in serum samples was assessed using Elecsys^®^ Anti-SARS-CoV-2 test (Roche Diagnostics, Mannheim, Germany), according to manufacturer’s instructions. Results are expressed as cut-off index (COI; signal of sample/cut-off); values ≥ 1.00 COI were considered positive.

### 2.8 Statistical analysis

Differences between groups were analysed with non-parametric (Mann-Whitney U, Wilcoxon signed rank) tests, as appropriate; non-parametric Spearman’s test was used to analyse correlations between variables; statistical analysis was performed with SPSS v27 (IBM, USA) and Prism v9.0 (GraphPad, USA) software packages. Differences were considered to be statistically significant when *p* value was < 0.05 (two-sided).

## 3 Results

### 3.1 Heterologous but not homologous anti-SARS-CoV-2 vaccinal setting induces persisting perturbation of CD16 expression and Ab-dependent functions in mature circulating NK cells

**Figure 1:**
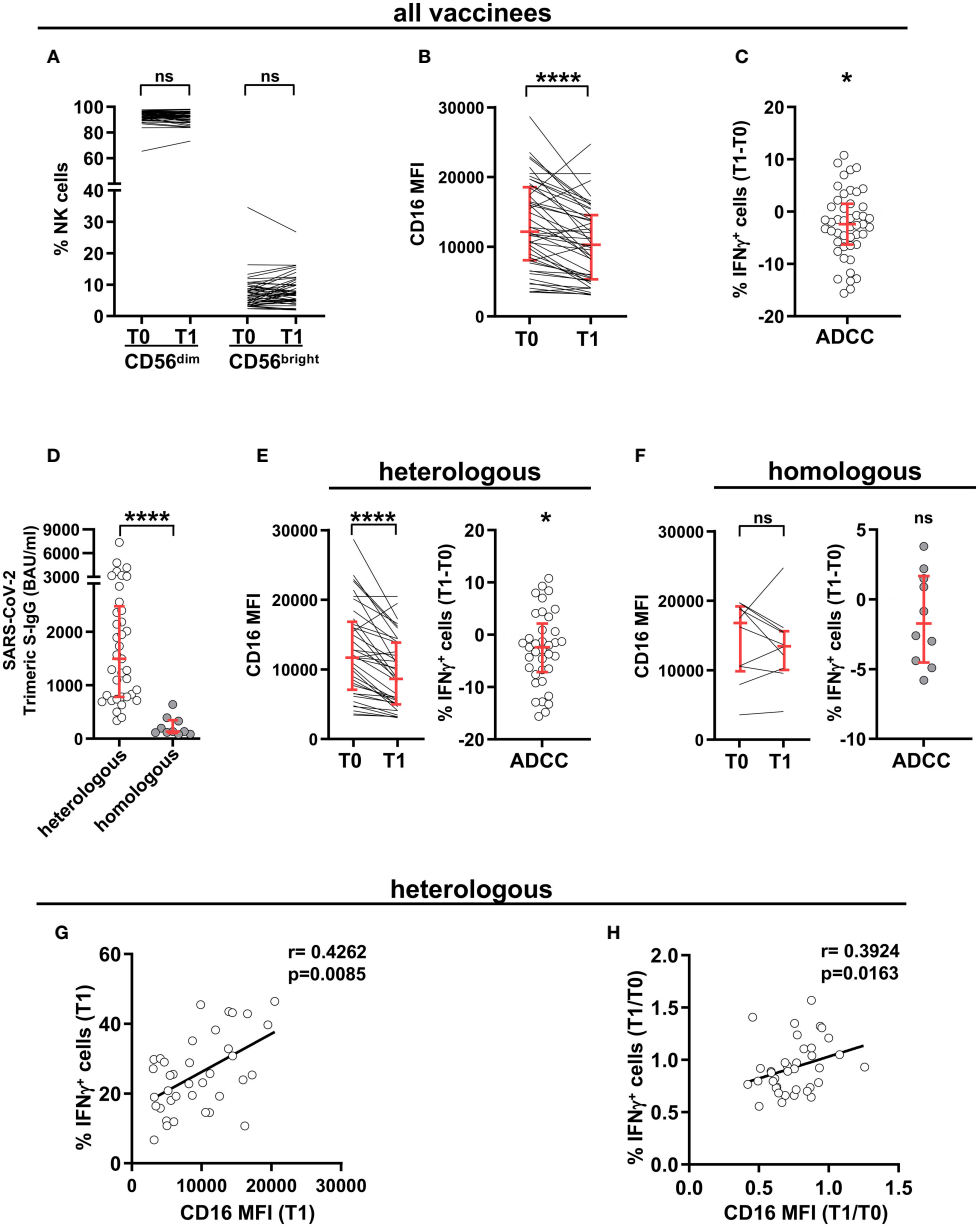
SARS-CoV-2 heterologous vaccination promotes long-term CD16 downregulation and reduced Ab-dependent IFNγ production. CD16 expression levels dictate post-vaccinal IFNγ production capability. PBMCs from 47 healthy individuals were analysed at pre-vaccination (T0) and 8 wk post-boost (T1) time points. **(A)** Percentage of peripheral blood CD56^dim^ and CD56^bright^ populations within CD3^-^CD56^+^ NK cells. **(B)** CD16 expression in CD3^-^CD56^dim^CD16^+^ NK cells. Data are expressed as median fluorescence intensity (MFI). **(C)** The difference (T1-T0) of the percentage of IFNγ-producing cells in CD56^dim^ NK cells was evaluated following PBMC stimulation with rituximab-opsonised Raji target (ADCC). **(D)** Anti-SARS-CoV-2 trimeric Spike IgG (binding antibody units, BAU) levels at T1 time point in subjects receiving heterologous (ChAdOX-1-S/BNT162b2, white circles, *n*=37) or homologous (ChAdOX-1-S/ChAdOX-1-S, grey circles, *n*=10) vaccination scheme. **(E, F)** CD16 median fluorescence intensity (MFI) (left panels) and difference (T1-T0) of the percentage of IFNγ-producing CD56^dim^ NK cells following PBMC stimulation with rituximab-opsonised Raji target (ADCC) (right panels), in heterologous **(E)** and homologous **(F)** vaccination settings. *p* values of pairwise and of “one sample” comparisons were calculated with Wilcoxon or Mann-Whitney non-parametric tests, as appropriate. Bars represent median with interquartile range. **(G, H)** Correlation analyses on heterologous vaccine-receiving individuals depicting the percentage of IFNγ^+^ cells and CD16 median fluorescence intensity (MFI) in post-vaccinal CD56^dim^ NK cells **(G)**, and the T1/T0 ratio of the percentage of IFNγ^+^ CD56^dim^ NK cells with the T1/T0 ratio of CD16 median fluorescence intensity (MFI) **(H)**. (*) <0.05, (****) <0.0001, (ns) not significant. (r) Spearman’s correlation coefficient.

The ability of vaccination to affect NK compartment has been noted in several immunization regimens (46). We evaluated the phenotypic and functional asset of peripheral blood mature (CD56^dim^) NK cells in a cohort of young adults, before the first cycle of vaccination with ChAdOx1-S adenoviral vector (T0) and 8 weeks after boost either with ChAdOx1-S or with BNT162b2 mRNA vaccine (T1). Although the abundance of CD56^dim^ (and of CD56^bright^) NK cells remained unchanged (**Figure 1A**), CD16 expression was markedly decreased after boost (**Figure 1B**). Importantly, the capability of CD56^dim^ NK cells to produce IFNγ when stimulated with mAb-opsonised target cells in the ADCC setting resulted significatively reduced at post-vaccination time point (**Figure 1C**), suggesting that receptor surface levels dictate NK cell functional response.

We took advantage of the coexistence of two different vaccinal regimens in our cohort of vaccinees. As already reported (50), heterologous ChAdOx1-S/BNT162b2 scheme induced distinctly higher anti-Spike antibody response at T1, as compared to homologous ChAdOx1-S/ChAdOx1-S combination (**Figure 1D**). Notably, CD16 downregulation and the consequent impairment of IFNγ production selectively characterized individuals that received heterologous, but not homologous, vaccinal scheme (**Figures 1E, F**, respectively), suggesting that the amplitude of the vaccinal antibody response may influence long-term CD16 expression *in vivo*.

Indeed, in vaccinees receiving adeno-mRNA heterologous vaccinal scheme, post-boost CD16 intensity positively correlated with the frequency of IFNγ-producing CD56^dim^ NK cells (**Figure 1G**); and the extent of CD16 and IFNγ response downregulation were significatively linked, when analyzed at the individual level (**Figure 1H**).

**Figure 2:**
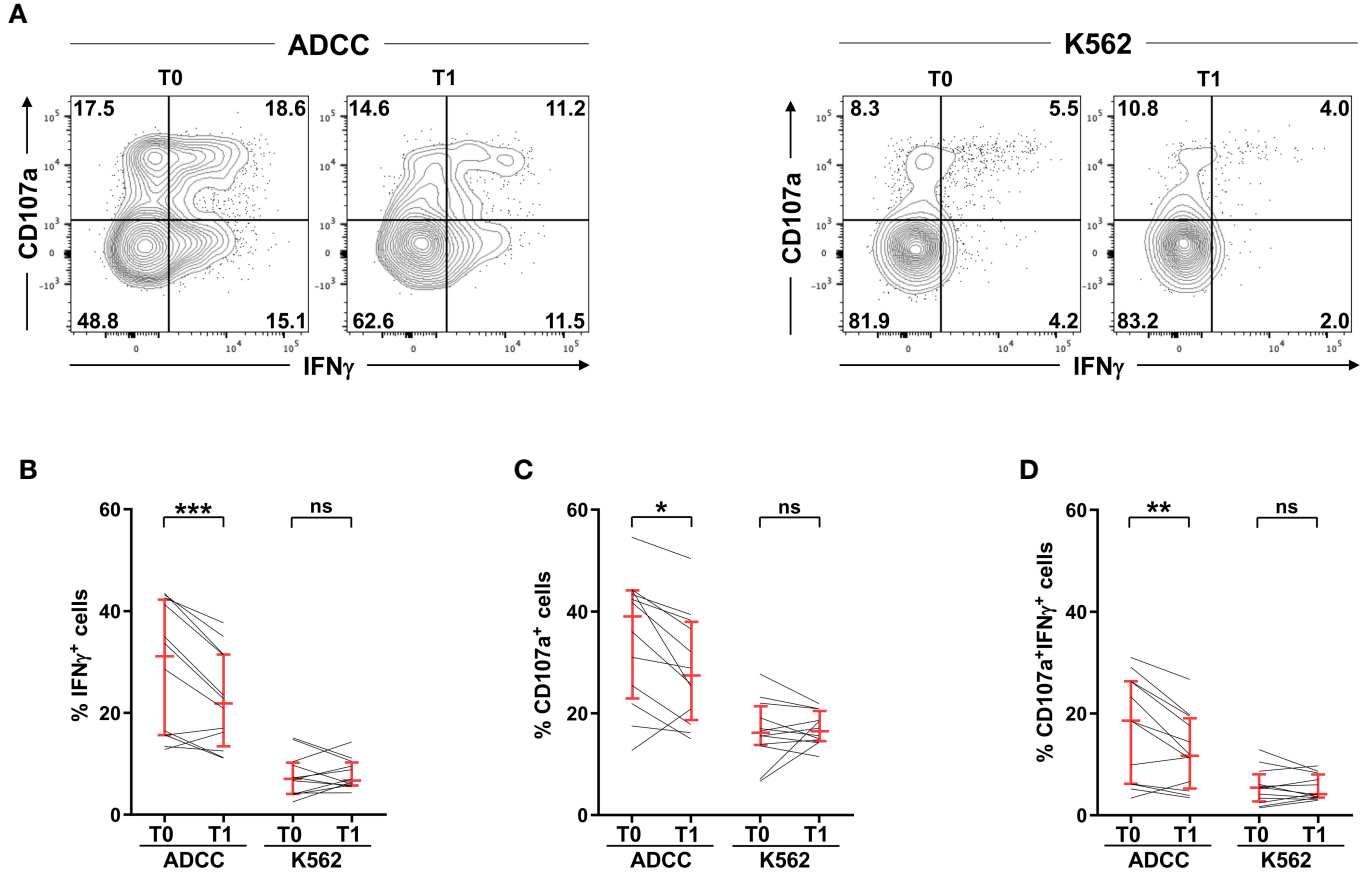
SARS-CoV-2 heterologous vaccination associates with the impairment of antibody-dependent, but not natural, effector functions. PBMCs from 12 vaccinees (heterologous adeno/mRNA vaccine setting) were stimulated with rituximab-opsonised Raji (ADCC) or K562 cell lines, pre- (T0) and post- (T1) vaccination. **(A)** Plots of a representative individual. Numbers represent percentage of gated cells; gate position was determined on a negative control sample (NK cells without target). The percentage of IFNγ^+^ **(B)**, CD107a^+^ **(C)** and IFNγ^+^CD107a^+^ **(D)** CD3^-^CD56^dim^ NK cells, was evaluated. Bars represent median with interquartile range. *p* values of pairwise comparisons were calculated with Wilcoxon non-parametric test. (*) <0.05, (**) <0.01, (***) <0.005, (ns) not significant.

CD16 stands as the most powerful activating receptor on mature NK cells, able to trigger cytotoxic activity and cytokine production (14, 15). Post-vaccinal impairment of IFNγ production (**Figures 2A, B**, left) was accompanied by reduced release of cytotoxic granules (**Figures 2A, C**, left), and lower frequency of cells capable of simultaneously performing both effector functions (**Figures 2A, D**, left), when NK cells were stimulated in the ADCC setting. Of note, the functional impairment was strictly confined to CD16-triggered functions, as demonstrated by the unchanged cytokine production and killing activity stimulated by interaction with K562 prototypic natural cytotoxicity target (**Figures 2A–D**, right).

The selective susceptibility of CD16-triggered functions to post-vaccinal receptor downmodulation was underlined by the correlation of receptor expression levels with CD16-dependent degranulation and multifunctional responses (**Supplementary Figure 1A**), and not with K562-dependent functional outcomes (**Supplementary Figure 1B**), at the individual level.

### 3.2 Vaccine-induced impairment of Fc-dependent NK functions is cell subset-dependent

**Figure 3:**
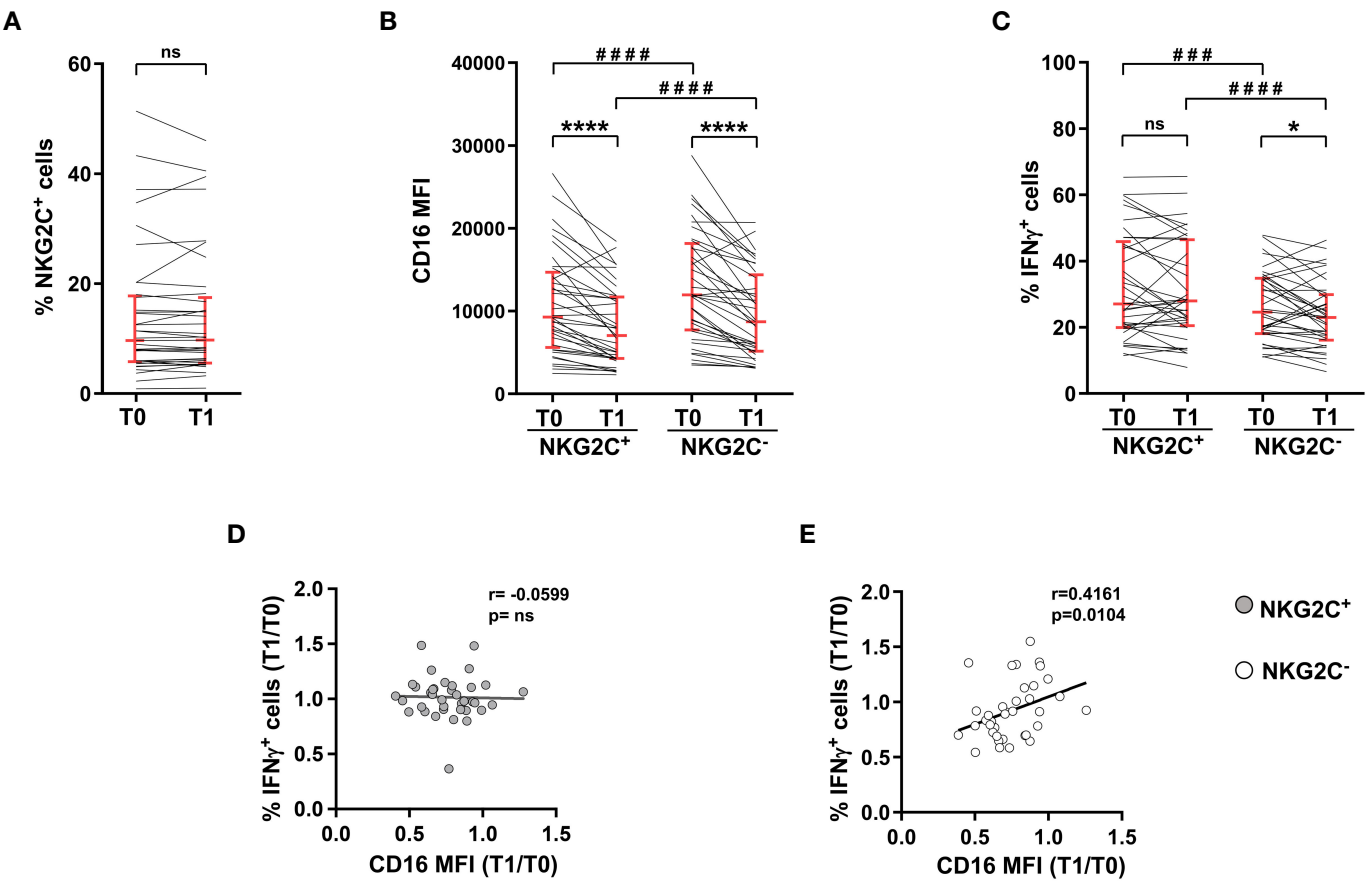
NKG2C^+^ NK cell-mediated IFNγ production is independent of CD16 post-vaccine downmodulation. Heterologous vaccine-receiving individuals were analyzed for: **(A)** percentage of NKG2C^+^ cells within CD56^dim^CD16^+^ NK cells; **(B)** CD16 surface levels (median fluorescence intensity, MFI) in NKG2C^+^ and NKG2C^-^ CD56^dim^CD16^+^ NK cells; **(C)** percentage of IFNγ-expressing NKG2C^+^ and NKG2C^-^ CD56^dim^ NK cells following PBMC stimulation with rituximab-opsonised Raji target (ADCC). Bars represent median with interquartile range. *p* values of pairwise comparisons were calculated with Wilcoxon non-parametric test (*n*=37). **(D, E)** Correlation between the T0/T1 ratio of the percentage of IFNγ^+^ cells and the T0/T1 ratio of CD16 median fluorescence intensity (MFI) in NKG2C^+^ (left panel) and NKG2C^-^ (right panel) CD56^dim^ NK cells. (*) <0.05, (###) =0.0005, (****,####) <0.0001, (ns) not significant, (r) Spearman’s correlation coefficient.

Peripheral blood mature NK cells are composed of different subsets, with distinct capabilities. The relative abundance of NKG2C^+^ CD56^dim^NK cells was highly heterogeneous in the cohort of young adults receiving Adeno/mRNA vaccination scheme, and it did not appreciably change between pre-vaccination and post-boost time points (**Figure 3A**). Although post-boost CD16 downregulation was comparably observed in both NKG2C^+^ and NKG2C^-^ NK cells, and despite the fact that NKG2C^+^ NK cells expressed CD16 with lower intensity either at baseline and after completion of the heterologous vaccination course (**Figure 3B**), they showed a more pronounced capability to produce IFNγ in response to CD16 engagement, with respect to NKG2C^-^ NK cells, at both time points. Indeed, post-vaccinal downmodulation of CD16 on NKG2C^+^ NK cells did not result in a functionally defective capability to produce IFNγ, differently from what observed on NKG2C^-^ counterpart (**Figure 3C**). Moreover, Ab-dependent functional response of NKG2C^+^ cells was insensitive to receptor downregulation, evaluated as T1 *vs* T0 ratio of CD16 intensity, as indicated by the absence of correlation between the two parameters (**Figure 3D**), in stark contrast with what shown for NKG2C^-^ NK cells (**Figure 3E**).

**Figure 4:**
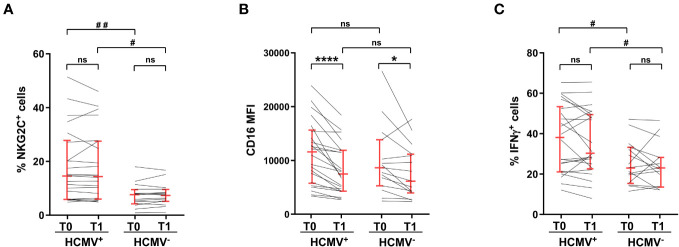
Hyperresponsiveness of NKG2C^+^ memory NK cells from HCMV-seropositive individuals to CD16 stimulation is unaffected by vaccination. **(A)** percentage of NKG2C^+^ cells within CD56dim NK cells, **(B)** CD16 surface levels (median fluorescence intensity, MFI) of NKG2C^+^CD56^dim^CD16^+^ NK cells, and **(C)** percentage of IFNγ-expressing NKG2C^+^ cells upon stimulation with rituximab-opsonised Raji target (ADCC), were evaluated in HCMV-seropositive (HCMV^+^, *n*=22) and HCMV-seronegative (HCMV^-^, *n*=15) individuals, pre- (T0) and post- (T1) heterologous vaccination. Bars represent median with interquartile range. *p* values of pairwise comparisons were calculated with Wilcoxon and Mann-Whitney non-parametric tests, as appropriate. (*,#) <0.05, (##) <0.005, (****) <0.0001, (ns) not significant.

The expansion of a NKG2C-expressing memory NK cell population, that is characterized by marked hyperresponsivity to CD16 stimulation, is associated to HCMV seropositive status (28, 29). Accordingly, HCMV^+^ vaccinees displayed a significatively larger population of NKG2C^+^CD56^dim^ NK cells, whose frequency was not perturbed by heterologous vaccine administration (**Figure 4A**). CD16 expression was significantly downmodulated on NKG2C^+^ cells of HCMV^+^ and HCMV^-^ individuals, although to a different extent (**Figure 4B**). In agreement with the literature (30–32, 34), NKG2C^+^ cells of HCMV^+^ donors showed a higher capability to produce IFNγ in response to stimulation with mAb-opsonised target cells, either before and after vaccine administration (**Figure 4C**). Strikingly, NKG2C^+^ cell capability to produce IFNγ was unaffected by post-vaccinal CD16 downmodulation, irrespectively of HCMV serostatus (**Figure 4C**), suggesting that the responsiveness of this peculiar NK cell subset is intrinsically more resistant to quantitative variations of the triggering receptor.

### 3.3 CD16 polymorphism affects post-vaccinal functional impairment: relevance of Fc affinity

**Figure 5:**
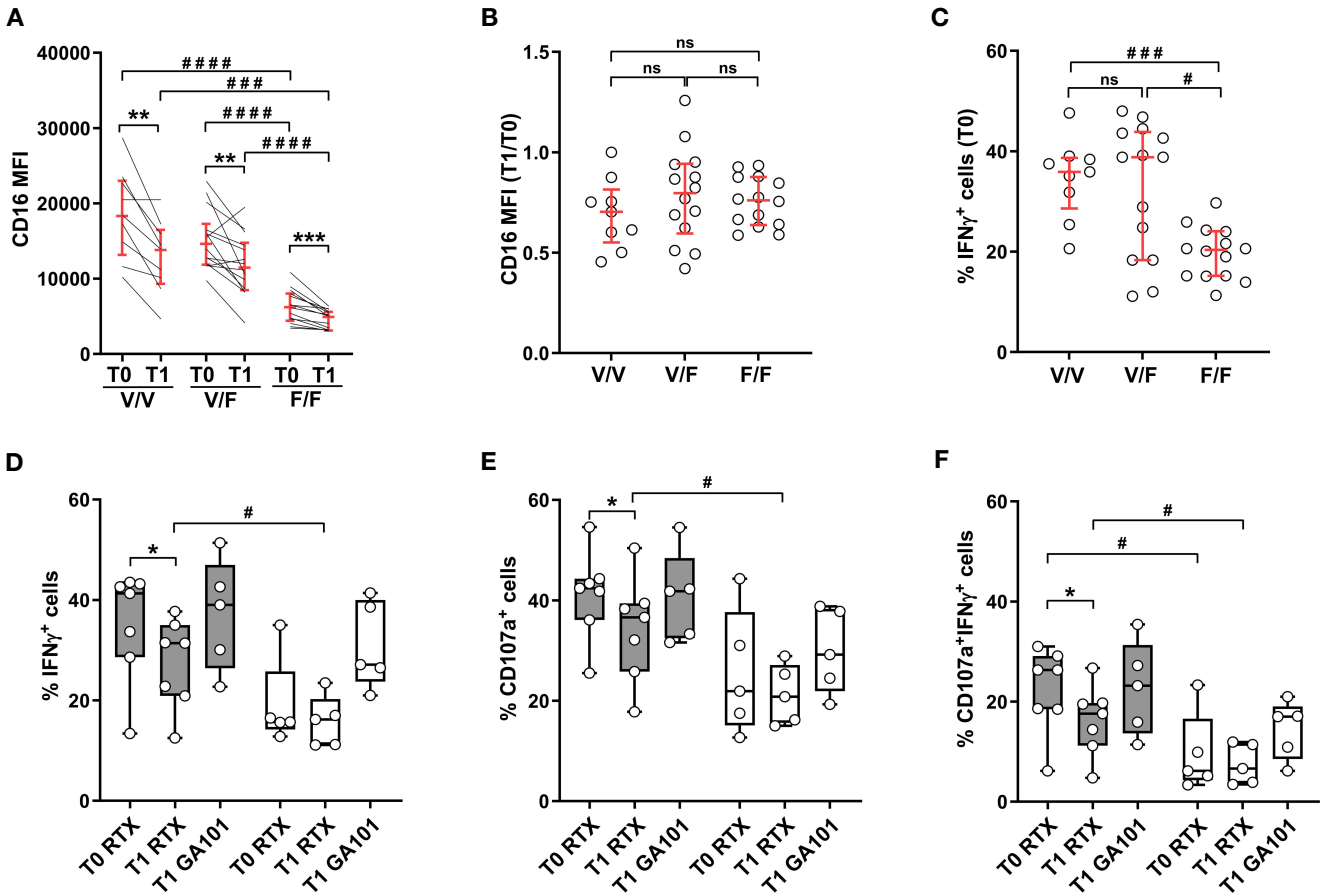
SARS-CoV-2 heterologous vaccination associates with CD16 downregulation independently of CD16/FcγRIIIA V158F polymorphism; allotype impact on antibody-mediated effector functions. Heterologous vaccine-receiving individuals were stratified by *FCGR3A* genotype (high affinity V/V homozygous, *n*=9; intermediate affinity V/F heterozygous, *n*=14; low affinity F/F homozygous, *n*=14). **(A)** CD16 expression levels (median fluorescence intensity, MFI) and **(B)** CD16 MFI T1/T0 ratio were analysed in CD56^dim^CD16^+^ cells. **(C)** Percentage of IFNγ^+^ CD56^dim^ NK cells was evaluated following PBMC stimulation with rituximab-opsonised Raji target (ADCC) at T0 time point. Percentage of IFNγ^+^ **(D)**, CD107a^+^ **(E)**, and IFNγ^+^CD107a^+^ **(F)** CD56^dim^ NK cells was evaluated following PBMC stimulation with rituximab (RTX)- or obinutuzumab (GA101)-opsonised Raji target. Genotyped individuals were grouped as follows: high affinity (V/V or V/F) variant-expressing (*n*=7, grey boxes) and low affinity (F/F) variant-expressing (*n*=5, white boxes). **(A–C)** Bars represent median with interquartile range; **(D–F)** Boxes extend from the 25th to 75th percentiles, whiskers go from the smallest value up to the largest. *p* values of pairwise comparisons were calculated with Wilcoxon or Mann-Whitney non-parametric tests, as appropriate, and reported when p<0.05. (*,#) <0.05, (**) <0.005, (***,###) <0.0005, (####) <0.0001, (ns) not significant.

Two allelic forms of human CD16 gene are defined by a single nucleotide polymorphism that, by changing the amino acid at position 158 (valine or phenylalanine), dictates receptor affinity for IgG ligand (23, 24). As shown in **Figure 5A**, CD16 expression levels were basally comparable in vaccinees either homozygous (158V/V) or heterozygous (158V/F) for the high affinity allele, and markedly lower in individuals homozygous for the low affinity allele (158F/F), as previously described (23); this expression pattern was conserved at post-vaccine time point. The significant diminution of CD16 expression at T1 was observed in all donors, irrespective of CD16 phenotype (**Figure 5A**), and at a roughly comparable extent, as evaluated by T1 *vs* T0 ratio of CD16 intensity (**Figure 5B**). We then analyzed the impact of CD16 genetic variants on Ab-dependent IFNγ production. At baseline (T0), NK cells from either 158V/V homozygous or 158V/F heterozygous individuals showed roughly comparable percentages of IFNγ-producing cells, while the response of 158F/F homozygous ones was distinctly lower (**Figure 5C**). However, while the donors bearing one or two copies of the high affinity allele showed a significant post-vaccinal impairment of CD16-triggered IFNγ production, cytotoxic granule release and capability to perform both effector functions, the response of individuals homozygous for the low-affinity 158F/F variant was unchanged (**Figures 5D–F**). Interestingly, stimulation with obinutuzumab (GA101) afucosylated anti-CD20 mAb, which binds CD16 with high affinity independently of genetic variants (41), compensated the defective response of 158F/F homozygous NK cells and also abrogated the post-vaccinal impairment of V/V and V/F donors (**Figures 5D–F**).

## 4 Discussion

Here we firstly describe that a marked downmodulation of CD16 receptor characterizes circulating, mature NK cells of individuals subjected to adenoviral/mRNA prime-boost anti-SARS-CoV-2 vaccinal regimen, in the absence of major perturbation of their differentiation status, evaluated as the frequency of CD56^bright^ and CD56^dim^ populations. The mechanisms are unknown, but a role for immune complexes formed by vaccinal antigen and vaccine-elicited IgG may be envisaged, considering that a persistent engagement of CD16 triggers receptor downmodulation by shedding and/or internalization (38, 39, 41, 42). Indeed, vaccinal Spike protein has been detected for an extended time period, either systemically and in secondary lymphoid organs, following administration of the mRNA vaccine formulation (44, 45). The observation that CD16 downmodulation significantly occurs only in the group of vaccinees that received the heterologous scheme (ChAdOx1-S/BNT162b2), that elicits higher average levels of anti-Spike Abs, and not in those receiving the less efficient ChAdOx1-S/ChAdOx1-S homologous vaccine, sustains our hypothesis. However, other distinctive features of the two vaccinal schemes, such as Ab post-translational modifications, or persistence of the antigen, may affect the generation and features of immune complexes, and, together with other immunologically relevant differences, may contribute to the different impact of vaccinal platforms on CD16 downmodulation.

The kinetics of post-vaccinal downmodulation of CD16 *in vivo* is unknown. Previous reports noted a drop of CD16^+^ NK cells shortly after SARS-CoV-2 vaccine boost (47, 48). Strikingly, we found a significantly reduced expression of the receptor 8 weeks after boosting. Of relevance, a similarly persisting *in vivo* CD16 downmodulation was reported in influenza virus-vaccinated individuals; in that context, *in vitro* immune complex-dependent receptor downmodulation needed a long time to revert (43).

We document here that CD16 downmodulation occurring in mRNA vaccine-boosted individuals bears important implications on the efficiency of Fc-dependent functions (12, 13), since these were found to markedly depend on receptor levels. Indeed, the frequency of NK cells able to degranulate, produce IFNγ or simultaneously perform both functions in response to IgG-opsonised targets was markedly reduced at post-boost time point. Importantly, this reduced functionality seems not to depend on a generalized state of exhaustion, as the effector functions induced by antibody-independent stimulation with a prototypic natural cytotoxicity target were conserved.

In the context of the growing appreciation of the Fc-dependent protective functions of vaccinal antibodies (3–9, 51), NK cells may play a special role. Indeed, the capability of polyfunctional Abs to trigger ADCC has been correlated with protection against SARS-CoV-2 variants of concern, across different vaccine platforms (3–9). In this regard, the persistent CD16 downregulation and hyporesponsiveness may heavily impact NK cell activation downstream of antibody responses, especially against new circulating virus variants.

Collectively taken, our data indicate that antibody-dependent NK cell effector responses may be dampened by post-vaccine CD16 downmodulation occurring in heterologous prime-boost setting; this phenomenon may constrain NK cell contribution to vaccinal protection.

Several evidence revealed that NK cell compartment is quantitatively and qualitatively perturbed upon vaccination (46). Our observation that CD16 downmodulation persisted long after completion of SARS-CoV-2 vaccination suggests that it may represent an example of long-term modifications of the innate immune compartment, named trained immunity (52, 53); related to this point, enduring antibody-dependent CD16 downregulation and hyperresponsiveness to low dose IL-15 have been observed in influenza virus vaccine recipients (43, 54).

Our data reveal that several host-dependent factors affect post-vaccinal CD16 functionality and thus modulate the capability of NK cells to work downstream of vaccinal antibody response.

CD16F158V polymorphism has been shown to be clinically relevant in different contexts (23–25), and it may play a role in antibody-mediated protection of vaccinees. Individuals homozygous for the low-affinity allele (F/F) display reduced levels of CD16 expression and functions, with respect to individuals expressing the high affinity variant (V/V and V/F), as previously shown (23, 24). Although receptor downregulation comparably occurred in vaccinees expressing either variant, F/F subjects were relatively resistant to post-vaccinal NK functional impairment and preserved their intrinsically lower responsivity. Although the mechanisms are unexplained, we previously demonstrated that sustained CD16 engagement in F/F NK cells results in a lower lysosomal degradation of CD16-associated ζ chain *in vitro*, thus presumably favoring the efficiency of signal transduction mechanisms upon CD16 internalization (41). The observation that stimulation with an afucosylated mAb counterbalances the post-vaccinal functional drop in high affinity variant-expressing (V/V and V/F) individuals, and the intrinsic lower functional capability of low affinity (F/F) homozygous subjects, suggests that the quality of antibodies, such as glycosylation profile, may influence the functional outcome at the individual level.

Our data highlight the peculiar behavior of a specific NK cell subset, marked by CD94/NKG2C expression, whose expansion and enhanced responsivity to CD16 engagement are driven by HCMV infection (28–34). Ab-dependent functions of NKG2C^+^ cells were unperturbed by post-boost CD16 downregulation, thus representing a further host-related factor that impacts the protective role played by NK cells downstream of vaccinal antibody production. Relevantly, the basal size of this population was previously found to correlate with SARS-CoV-2 vaccine efficacy, while the expansion of NKG2C^+^ cells correlated with anti-influenza virus vaccinal response (37).

These data thus identify important genetic and environmental factors that may modulate, in a host-dependent manner, NK cell contribution to antibody-mediated protection against disease.

Additionally, the correlation analysis at the individual level revealed that the preservation of CD16 expression, directly correlated with anti-Spike antibody titer in heterologous prime-boost vaccination setting (**Supplementary Figure 2**); this observation opens to the possibility that Fc-dependent NK cell functions may participate in the amplification of post-boost Ab production, by means of a rapid release of several immunoregulatory cytokines (14–16). On this line, it was reported that the augmentation of IFNγ-producing NK cells at recall correlated with SARS-CoV-2 mRNA vaccine outcome, in terms of antibody response durability and breadth against emerging virus variants (47). Relevantly, NK and CD8 T cells in draining LN were the source of IFNγ after secondary immunization with BNT162b2 mRNA vaccine (17).

In synthesis, by taking advantage of an age-homogeneous cohort of young individuals that underwent SARS-Cov2 heterologous vaccination regimen, our results show that the contribution of NK cells to vaccinal protection may be interfered by CD16 downregulation. The functional impact of reduced receptor expression displays a large interindividual variability, that depends on both vaccine-related and host-dependent factors (i.e. genotype, HCMV carrier status, expansion of NKG2C^+^ adaptive population), and that may also be modulated by the quality of vaccine-elicited antibodies, such as the profile of post-translational modifications. If generalized, this mechanism could bear wide-range implications on the clinical efficacy of distinct vaccine platforms and regimens.

## Data availability statement

The sequencing data presented in the study are deposited in the NCBI Sequence Read Archive database, with links to BioProject accession PRJNA1032283 in the NCBI BioProject database (https://www.ncbi.nlm.nih.gov/bioproject/).

## Ethics statement

The studies involving humans were approved by Ethics Committee of Sapienza University of Rome (EC identifier 6020 - Prot. 0486/2021). The studies were conducted in accordance with the local legislation and institutional requirements. The participants provided their written informed consent to participate in this study.

## Author contributions

CC: Data curation, Formal analysis, Investigation, Methodology, Validation, Writing – review & editing, Funding acquisition. DF: Data curation, Formal analysis, Investigation, Methodology, Validation, Writing – review & editing. DC: Formal analysis, Investigation, Methodology, Writing – review & editing. OT: Methodology, Validation, Writing – review & editing. AA: Validation, Writing – review & editing, Data curation. EA: Validation, Writing – review & editing, Data curation. GG: Methodology, Writing – review & editing, Validation. FB: Validation, Writing – review & editing, Methodology. RM: Investigation, Methodology, Writing – review & editing. DA: Conceptualization, Funding acquisition, Validation, Writing – review & editing. GP: Conceptualization, Formal analysis, Funding acquisition, Project administration, Supervision, Writing – original draft. RG: Conceptualization, Formal analysis, Funding acquisition, Project administration, Supervision, Writing – original draft.
