# Supplementary material for: Impact of SARS-CoV-2 vaccination on FcγRIIIA/CD16 dynamics in Natural Killer cells: relevance for antibody-dependent functions
Source: Front Immunol. 2023 Nov 16;14:1285203. doi: 10.3389/fimmu.2023.1285203 (PMC10693335; doi:10.3389/fimmu.2023.1285203)
Supplement: Supplementary file 1 [file DataSheet_1.pdf]

Supplementary Material

Impact of SARS-CoV-2 vaccination on FcγRIIIA/CD16 dynamics in Natural Killer cells: relevance for antibody-dependent functions

Cristina Capuano[1†], Davide De Federicis[2,3†], Daniel Ciuti[2], Ombretta Turriziani[3], Antonio Angeloni[2], Emanuela Anastasi[2], Giuseppe Giannini[3], Francesca Belardinilli[3], Rosa Molfetta[3], Domenico Alvaro[4], Gabriella Palmieri[2*] and Ricciarda Galandrini[2*]

[1]Departmental Faculty of Medicine and Surgery, UniCamillus-Saint Camillus International University of Health and Medical Sciences, Rome, Italy
[2]Department of Experimental Medicine, Sapienza University of Rome, Italy
[3]Department of Molecular Medicine, Sapienza University of Rome, Italy
[4]Department of Translational and Precision Medicine, Sapienza University of Rome, Italy

[†]These authors contributed equally to this work and share first authorship
*These authors contributed equally to this work and share last authorship

***Correspondence:**

Gabriella Palmieri
gabriella.palmieri@uniroma1.it

Ricciarda Galandrini
ricciarda.galandrini@uniroma1.it

Supplementary Table 1. **Characteristics of the vaccinee cohort**

N°	Age (years)	Sex	CD16 genotype	anti-N Abs (T0)	anti-N Abs (T1)	anti-HCMV IgG (T0)	Vaccination setting
**1**	23	female	F/F	neg	neg	pos	Heterologous[a]
**2**	28	male	F/F	neg	neg	neg	Heterologous
**3**	27	male	V/V	neg	neg	pos	Heterologous
**4**	27	female	V/V	neg	neg	pos	Heterologous
**5**	27	female	F/V	neg	neg	pos	Heterologous
**6**	26	male	V/V	neg	neg	pos	Heterologous
**7**	21	female	F/V	pos	pos	pos	Heterologous
**8**	23	female	V/V	pos	pos	neg	Heterologous
**9**	26	male	F/F	neg	neg	neg	Heterologous
**10**	28	male	F/V	neg	neg	pos	Heterologous
**11**	26	male	F/V	neg	neg	neg	Homologous[b]
**12**	25	female	F/F	neg	neg	pos	Heterologous
**13**	27	female	F/F	neg	neg	neg	Heterologous
**14**	26	female	F/V	neg	neg	pos	Homologous
**15**	26	male	F/V	neg	neg	pos	Heterologous
**16**	25	female	F/V	neg	neg	pos	Homologous
**17**	25	male	F/F	neg	neg	pos	Heterologous
**18**	24	female	F/V	neg	neg	pos	Heterologous
**19**	29	female	F/V	neg	neg	pos	Homologous
**20**	25	female	F/V	pos	pos	neg	Heterologous
**21**	26	female	V/V	neg	neg	neg	Heterologous
**22**	27	female	F/V	neg	neg	pos	Homologous
**23**	34	male	F/V	neg	neg	pos	Heterologous
**24**	26	male	F/V	neg	neg	pos	Homologous
**25**	29	female	F/V	neg	neg	pos	Heterologous
**26**	26	female	F/V	neg	neg	neg	Heterologous
**27**	24	male	V/V	neg	pos	neg	Heterologous
**28**	28	male	F/F	neg	neg	neg	Heterologous
**29**	26	female	F/F	neg	neg	pos	Heterologous
**30**	26	female	F/V	neg	neg	pos	Homologous
**31**	26	female	F/F	neg	neg	pos	Heterologous
**32**	27	female	F/F	neg	neg	neg	Heterologous
**33**	29	female	F/V	neg	neg	neg	Homologous
**34**	24	male	F/V	neg	neg	neg	Heterologous
**35**	25	female	V/V	neg	neg	pos	Heterologous
**36**	26	male	F/V	neg	neg	pos	Heterologous
**37**	25	female	V/V	neg	neg	pos	Heterologous
**38**	25	male	F/F	neg	neg	pos	Heterologous
**39**	24	female	F/F	neg	neg	neg	Heterologous
**40**	27	female	F/V	neg	neg	neg	Homologous
**41**	27	male	V/V	neg	neg	neg	Heterologous
**42**	28	male	F/V	neg	neg	neg	Heterologous
**43**	24	female	F/F	neg	neg	pos	Heterologous
**44**	28	male	F/V	neg	neg	neg	Homologous
**45**	24	male	F/V	pos	pos	pos	Heterologous
**46**	26	female	F/V	neg	neg	neg	Heterologous
**47**	28	female	F/F	neg	neg	pos	Heterologous

[a]ChadOx1-S/BNT16b2 heterologous vaccinal scheme

[b]ChadOx1-S/ChadOx-1S homologous vaccinal scheme

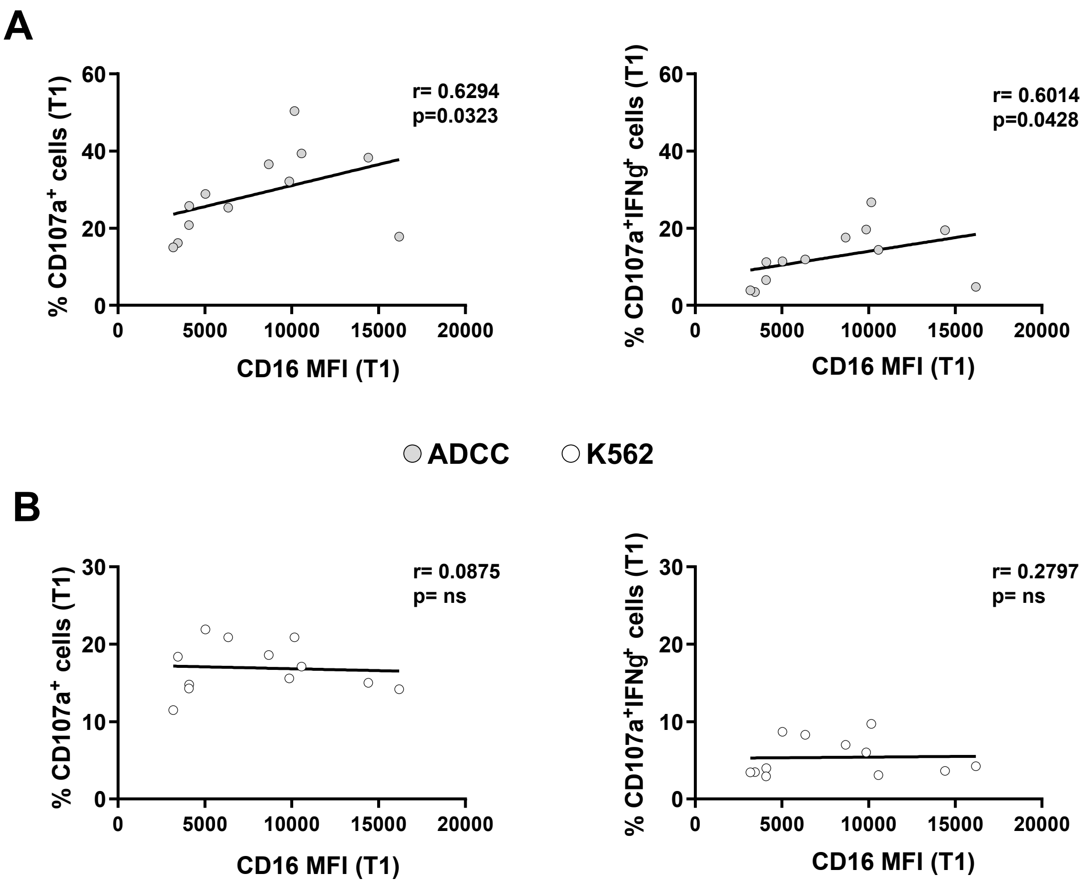

Supplementary Figure 1. CD16 expression levels correlate with antibody-dependent, but not natural, effector functions following heterologous SARS-CoV-2 vaccination. Correlation between CD16 median fluorescence intensity (MFI) at T1 time point and the percentage of CD107a$^+$ cells (left panels) and CD107a$^+$IFNγ$^+$ cells (right panels) in CD56^{dim} NK cells (*n*=12), following PBMC stimulation with rituximab-opsonised Raji (grey circles) **(A)** or K562 (white circles) cell lines **(B)**. (r) Spearman's correlation coefficient, (*) <0.05, (ns) not significant.

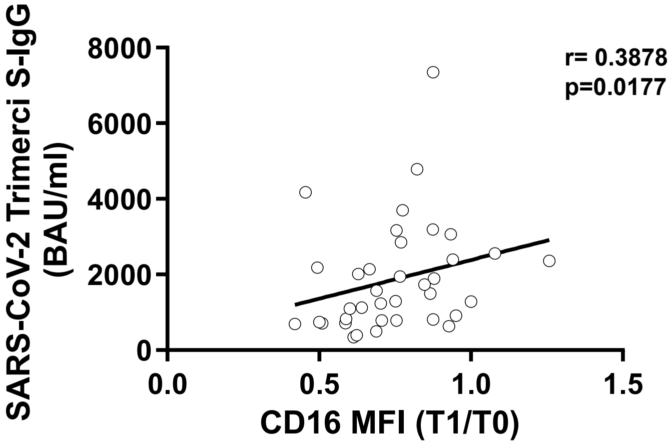

Supplementary Figure 2. Post-vaccinal persistence of CD16 expression correlates with vaccinal antibody response.

Correlation between anti-SARS-CoV-2 trimeric Spike IgG and the T1/T0 ratio of CD16 median fluorescence intensity (MFI) in heterologous (ChAdOx1-S/BNT162b2, *n*=37) vaccination setting. (r) Spearman's correlation coefficient, (*) <0.05.
